# Remission Depth in Metastatic Hormone-Sensitive Prostate Cancer Is Associated With Prognosis in Patients With Initial Prostate-Specific Antigen Values Above 100 Ng/ML

**DOI:** 10.7759/cureus.54036

**Published:** 2024-02-11

**Authors:** Takeshi Azuma, Akimasa Katsumata, Masato Kano, Koji Tsumura

**Affiliations:** 1 Urology, Tokyo Metropolitan Tama Medical Center, Tokyo, JPN

**Keywords:** prognosis, hormonal therapy, remission depth, prostate-specific antigen, metastatic hormone-sensitive prostate cancer

## Abstract

Introduction: Recently, new drugs have caused a paradigm shift in the treatment of metastatic hormone-sensitive prostate cancer (HSPC). Meanwhile, research has identified several prognostic factors of metastatic HSPC.

Objective: The present study focused on remission depth in metastatic HSPC and evaluated its association with remission depth.

Method: We analyzed 427 patients diagnosed with metastatic HSPC with serum initial prostate-specific antigen (PSA) > 100 ng/ml. The nadir serum PSA value was used as a marker of remission depth for each duration to castration resistance by using receiver operating characteristic (ROC) curves. Cox proportional hazards regression was used to assess for any correlation of progression-free survival (PFS) and overall survival (OS) with the nadir PSA level.

Results: The cut-off value for the nadir PSA level per time to castration resistance (TTCR) at three, five, seven, and nine years was calculated. The nadir PSA value alone was able to predict prognosis because of its high sensitivity, high specificity, and high AUC in ROC analysis. The nadir PSA level can be an independent prognostic marker not only for TTCR but also for OS on multivariate analysis.

Conclusion: We identified the cut-off value for nadir PSA per TTCR period in patients with metastatic HSPC. The nadir PSA value alone can predict prognosis; this demonstrates utility in routine clinical practice due to its simplicity and accuracy.

## Introduction

Treatment for castration-resistant prostate cancer (CRPC) has progressed over the past several years. Newly developed drugs have caused a paradigm shift in the treatment of metastatic hormone-sensitive prostate cancer (HSPC) [1-5]. Phase III clinical trials have shown that abiraterone, apalutamide, enzalutamide, and docetaxel are effective in improving the prognosis of metastatic HSPC [1-6].

The search for prognostic factors of metastatic HSPC has been proceeding in parallel with these developments and several prognostic markers have been identified [7-11]. One of these prognostic markers [12-14] is the nadir prostate-specific antigen (PSA) level. Previous studies examining the serum nadir PSA level used various cut-off values, with most using 0.2 ng/ml, the conventional cut-off value [15,16]. However, the reason for choosing this value was never made explicit.

Remission depth is an important prognostic factor in patients with hematological malignancies [17]. Achieving a deep response can lead to a good clinical prognosis [18-20]. The current methods of assessing the prognosis of hematological malignancies include polymerase chain reaction (PCR) analysis, which can be used to assess the treatment effect in patients with chronic myeloid leukemia (CML) in routine clinical practice. PCR analysis is highly sensitive and is associated with the prognosis of CML, although, in patients with epithelial-derived malignancies, a serum tumor biomarker might be more useful as a marker of remission depth. However, serum tumor biomarkers have low sensitivity for most epithelial-derived malignancies, which differ from hematologic malignancies in lacking a useful method of detecting fine, residual cancer cells, i.e., remission depth. Only prostate cancer has a highly sensitive serum tumor biomarker, PSA [21]. For example, the serum PSA level immediately after a radical prostatectomy reflects residual prostate cancer, and the serum nadir PSA value is useful as a post-prostatectomy prognostic marker [22]. The present study examined the use of the nadir serum PSA level as a marker of remission after treatment. 

The present study assessed the association between prognosis and remission depth in metastatic HSPC by analyzing the serum nadir PSA cut-off level as a remission depth marker for time to castration resistance (TTCR) by using receiver operating characteristic (ROC) curves. 

## Materials and methods

Patients

Of 542 patients with iPSA > 100 ng/ml who were initially treated at Tokyo Metropolitan Tama Medical Center between April 1st, 2002 and March 31st, 2021, 427 had at least one metastasis. These 427 patients were eligible for this study and enrolled, and their follow-up data were retrieved from the hospital medical records between June 1st and July 31st, 2022. Authors had access to information that could identify individual participants during or after data collection. The present retrospective study was performed in compliance with the ethical standards of the Declaration of Helsinki and was approved by the ethics review board at Tokyo Metropolitan Tama Medical Center (4-3). Comprehensive informed consent was obtained from all the participants. 

Treatment protocol

The patients had received androgen-deprivation therapy with or without irradiation against bone metastases. CRPC was defined as two consecutive increases in PSA or a clear, radiological finding of progressive disease. After diagnosis of CRPC, patients received systemic chemotherapy (docetaxel and/or cabazitaxel) and/or second-generation androgen deprivation therapy.

Data

The following data were collected from the medical records: age; initial PSA; nadir PSA; hemoglobin; LDH; ALP; total Gleason score; metastasis site; EOD; and treatment history.

Statistics

The nadir PSA cut-off value was determined using ROC curve analysis. PFS was defined as the time from diagnosis to biochemical or radiological failure. OS was defined as the time from diagnosis to death from any cause. The distribution of PFS and OS was mapped using the Kaplan-Meier method. Cox proportional hazards regression analysis was performed to analyze independent predictors of PFS and OS. All statistical analyses were performed using the JMP® software package (SAS Institute, Cary, NC), and p <0.05 was considered to indicate statistical significance.

## Results

Patient characteristics

**Figure 1:**
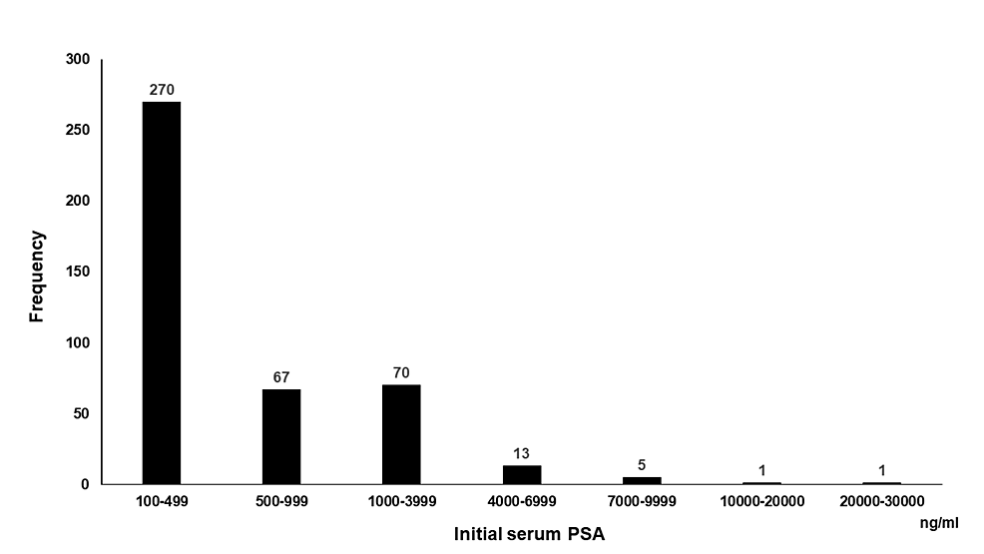
Distribution of initial PSA value. PSA, prostate-specific antigen

Table 1 shows the patient characteristics. Patients with a Gleason score ≧8, bone metastasis, lung metastasis, and liver metastasis numbered 364 (85.2%), 316 (74.0%), 46 (10.8%), and nine (2.1%), respectively. Based on the LATITUDE trial and CHAARTTED trial, 248 (58.1%) patients had high-risk HSPC, and 250 (58.5%) had high-volume HSPC, respectively. Figure 1 shows the distribution of the serum initial prostate-specific antigen (iPSA) values. The median iPSA value was 298.5 ng/ml, and iPSA≧1000 ng/ml was found in 90 (21.1%) patients (Figure 1). The median follow-up period was 39.6 months, during which 273 (63.9%) patients died. The median follow-up period for the 154 (36.1%) surviving patients was 37.0 months. The median OS was 58.8 months. The OS rate at three, five, seven, and nine years was 65.2, 49.7, 36.3, and 27.6%, respectively.

**Table 1:** Patient characteristics (n=427) PSA, prostate-specific antigen

Patient Characteristics		
Age (in years)	Average	75.4
	Range	44-96
		number of patients (%)
Prostate-specific antigen	100-999	337 (79.5)
	1000-9999	88 (20.0)
	10000-30000	2 (0.5)
Total Gleason score	7	63 (14.8)
	8	157 (36.8)
	9	183 (42.9)
	10	24 (5.5)
Metastasis site	Regional lymph node	334 (78.2)
	Distant Lymph node	163 (38.2)
	Bone	316 (74.0)
	Lung	46 (10.8)
	Liver	9 (2.1)
CHAARTED	Low	177 (41.5)
	High	250 (58.5)
LATITUDE	Low	179 (41.9)
	High	248 (58.1)
PSA nadir	<0.5	217 (50.8)
	<0.2	162 (37.9)
	<0.1	126 (29.5)
	<0.05	106 (24.8)
	<0.02	82 (19.2)
Primary treatment	Androgen deprivation therapy	413 (97.0)
	Abiraterone	2 (0.5)
	Enzalutamide	2 (0.5)
	Apalutamide	7 (1.5)
	Docetaxel	2 (0.5)
Post-CRPC treatment	Abiraterone	54 (12.6)
	Enzalutamide	73 (17.1)
	Docetaxel	78 (18.3)
	Cabazitaxel	9 (2.1)
Median laboratory value at diagnosis	Hemoglobin	12.9
	Lactate dehydrogenase	194
	Alkaline phosphatase	320
	Albumin	4

ROC analysis

**Figure 2:**
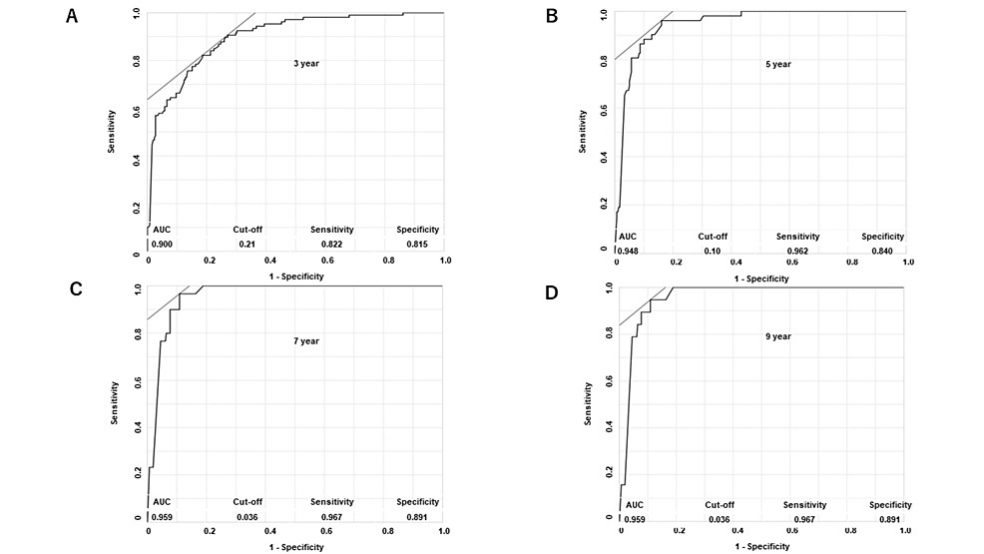
ROC curve analysis of nadir PSA value for each TTCR (3 (A), 5 (B), 7 (C), and 9 (D) years). PSA, prostate-specific antigen; AUC, under the curve

The nadir PSA cut-off value for each TTCR (three, five, seven, and nine years) was determined by ROC curve analysis (Figure 2). At the nadir PSA cut-off value of 0.21, 0.10, 0.036, and 0.036 ng/ml for three-, five-, seven-, and nine-year TTCR, the sensitivity was 82.2%, 96.2%, 96.7%, and 96.7%, and the specificity was 81.5%, 84.0%, 89.1%, and 89.1%, respectively. The area under the ROC curve was 90.0%, 94.8%, 95.9%, and 95.9% for the three-, five-, seven-, and nine-year TTCR, respectively.

Correlation analysis

Table 2 shows the results of our analysis of the correlation between several factors and nadir PSA. All the nadir PSA cut-off values correlated with initial PSA, extent of disease (EOD) score, hemoglobin, albumin, lactate dehydrogenase, alkaline phosphatase, and the CHAARTED and LATITUDE criteria.

**Table 2:** The correlation between several factors and nadir PSA P< 0.05 was considered statistically significant. PSA, prostate-specific antigen; EOD, extent of disease; LDH, lactate dehydrogenase;  ALP, alkaline phosphatase; CHAARTED, chemohormonal therapy versus androgen ablation randomized trial for extensive disease in prostate cancer

The correlation between several factors and nadir PSA												
		Nadir PSA				Nadir PSA				Nadir PSA		
		≥0.21	<0.21	p-value		≥0.10	<0.10	p-value		≥0.036	<0.036	p-value
Age	<65 yrs	26 (6.1%)	18 (4.2%)	0.88	<65 yr	33 (7.7%)	11 (2.6%)	0.73	<65 yr	28 (6.6%)	16 (3.8%)	0.47
	≥65 yrs	231 (54.1%)	152 (35.6%)		≥65 yr	296 (69.3%)	87 (20.4%)		≥65 yr	264 (61.8%)	119 (27.9%)	
Initial PSA	<300 ng/ml	117 (27.4%)	98 (23.0%)	0.014	<300 ng/ml	155 (36.3%)	60 (14.1%)	0.014	<300 ng/ml	132 (30.9%)	83 (19.4%)	0.002
	≥300 ng/ml	140 (32.8%)	72 (16.9%)		≥300 ng/ml	174 (40.8%)	38 (8.9%)		≥300 ng/ml	160 (37.5%)	52 (12.2%)	
Gleason score	<9	138 (32.3%)	83 (19.4%)	0.32	<9	175 (50.0%)	46 (10.8%)	0.27	<9	155 (36.3%)	66 (15.5%)	0.42
	≥9	119 (27.9%)	87 (20.4%)		≥9	154 (36.1%)	52 (12.2%)		≥9	137 (32.1%)	69 (16.2%)	
EOD	<2	104 (24.4%)	116 (27.2%)	<0.001	<2	148 (34.7%)	72 16.9%)	<0.001	<2	121 (28.3%)	99 (23.2%)	<0.001
	≥2	153 (35.8%)	54 (12.7%)		≥2	181 (42.4%)	26 (6.1%)		≥2	171 (40.1%)	36 (8.4%)	
Hemoglobin	>12 g/dl	147 (34.4%)	137 (32.1%)	<0.001	>12 g/dl	201 (47.1%)	83 (19.4%)	<0.001	>12 g/dl	175 (41.0%)	109 (25.5%)	<0.001
	≤12 g/dl	110 (25.8%)	33 (7.7%)		≤12 g/dl	128 (30.0%)	15 (3.51%)		≤12 g/dl	117 (27.4%)	26 (6.1%)	
Albumin	>3.9 g/dl	125 (29.3%)	114 (26.7%)	<0.001	>3.9 g/dl	164 (38.4%)	75 (17.6%)	<0.001	>3.9 g/dl	143 (33.5%)	96 (22.5%)	<0.001
	≤3.9 g/dl	132 (30.9%)	56 (13.1%)		≤3.9 g/dl	165 (38.6%)	23 (5.4%)		≤3.9 g/dl	149 (34.9%)	39 (9.1%)	
LDH	≤250 U/l	193 (45.2%)	149 (34.9%)	0.001	≤250 U/l	252 (59.0%)	90 (21.0%)	<0.001	≤250 U/l	218 (51.1%)	124 (29.0%)	<0.001
	>250 U/l	64 (15.0%)	21 (4.9%)		>250 U/l	77 (18.0%)	8 (1.9%)		>250 U/l	74 (17.3%)	11 (2.6%)	
ALP	≤500 U/l	156 (36.5%)	134 (31.4%)	<0.001	≤500 U/l	208 (48.7%)	82 (19.2%)	<0.001	≤500 U/l	180 (42.2%)	110 (25.8%)	<0.001
	>500 U/l	101 (23.7%)	36 (8.4%)		>500 U/l	121 (28.3%)	16 (3.8%)		>500 U/l	112 (26.2%)	25 (5.9%)	
Lung metastasis	No	229 (53.6%)	152 (35.6%)	0.92	no	293 (68.6%)	88 (20.6%)	0.84	no	259 (60.’%)	122 (28.6%)	0.60
	yes	28 (6.3%)	18 (4.2%)		yes	36 (8.4%)	10 (2.3%)		yes	33 (7.7%)	13 (3.0%)	
Liver metastasis	No	253 (59.3%)	165 (38.6%)	0.33	no	322 (75.4%)	96 (22.5%)	0.96	No	287 (67.2%)	131 (30.7%)	0.40
	Yes	4 (0.9%)	5 (1.2%)		yes	7 (1.6%)	2 (0.5%)		Yes	5 (1.2%)	4 (0.9%)	
CHAARTED	Low volume	76 (17.8%)	101 (23.7%)	<0.001	Low-volume	108 (25.3%)	69 (16.2%)	<0.001	Low volume	87 (20.4%)	90 (21.1%)	<0.001
	High volume	181 (42.4%)	69 (16.2%)		High-volume	221 (51.8%)	29 (6.8%)		High volume	205 (48.0%)	45 (10.5%)	
LATITUDE	Low risk	86 (20.1%)	100 (23.4%)	<0.001	Low-risk	119 (27.9%)	67 (15.7%)	<0.001	Low risk	98 (23.0%)	88 (20.6%)	<0.001
	High risk	171 (40.1%)	70 (16.4%)		High-risk	210 (49.2%)	31 (7.3%)		High risk	194 (45.4%)	47 (11.0%)	

Survival analysis

**Figure 3:**
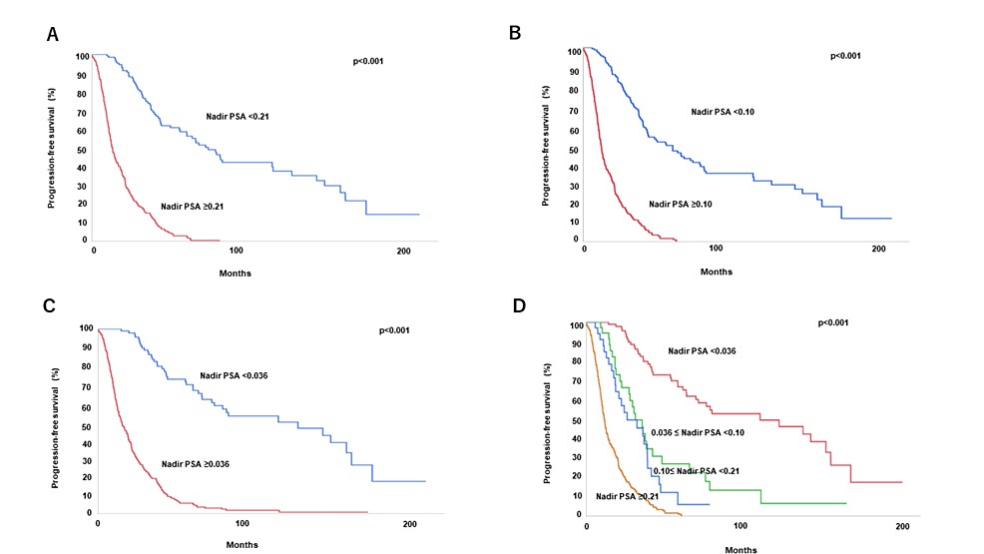
Kaplan-Meier survival curves for OS stratified by nadir PSA value. The cutoff values were 0.21 (A), 0.10 (B), and 0.036 ng/ml (C). Kaplan-Meier survival curves for PFS stratified by nadir PSA value(D). PSA, prostate-specific antigen

**Figure 4:**
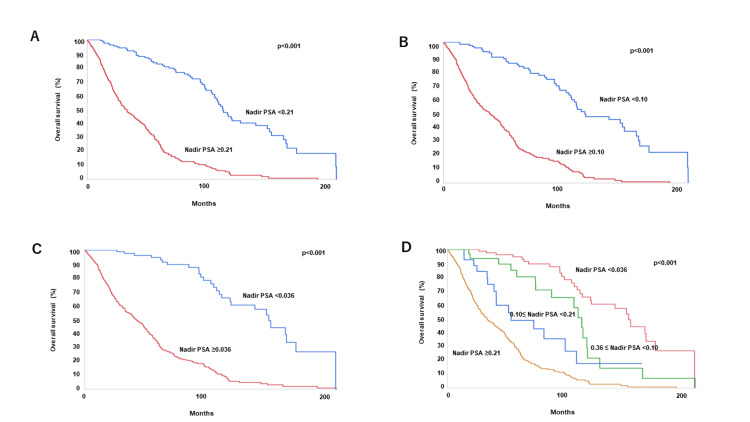
Kaplan-Meier survival curves for OS divided by nadir PSA value. The cutoff values were 0.21 (A), 0.10 (B), and 0.036 ng/ml(C). Kaplan-Meier survival curves for OS stratified by nadir PSA value (D). PSA, prostate-specific antigen

Kaplan-Meier survival analysis was used to analyze progression-free survival (PFS) and overall survival (OS) by nadir PSA value. The three-year PFS rate for those with nadir PSA <0.21, the five-year PFS rate for those with nadir PSA <0.10, the seven-year PFS rate for those with nadir PSA <0.036, and the nine-year PFS rate for those with nadir PSA <0.036 was 67.7, 66.9, 63.0, and 52.3 %, respectively (Figures 3A-C). Figure 3D shows the PFS curve after dividing the four groups by the nadir PSA value. The median PFS for nadir PSA ≥0.21, 0.10≤ nadir PSA <0.21, 0.036 ≤ nadir PSA <0.10, and nadir PSA <0.036 was 11.8, 26.3, 30.9, and 111.5 months, respectively. The three-year OS rate for the group with nadir PSA <0.21, the five-year OS rate for nadir PSA <0.10, the seven-year OS rate for nadir PSA <0.036, and the nine-year OS rate for nadir PSA <0.036 was 93.3, 82.7, 89.6, and 75.8 %, respectively (Figures 4A-C). Figure 4D shows the OS curve after dividing the four groups by the nadir PSA value. The median OS for nadir PSA ≥0.21, 0.10≤ nadir PSA <0.21, 0.036 ≤ nadir PSA <0.10, and nadir PSA <0.036 was 34.2, 52.9, 113.4, and 157.3 months, respectively.

Univariate and multivariate analyses

Univariate analysis revealed that the nadir PSA value, hemoglobin, albumin, lactate dehydrogenase, alkaline phosphatase, EOD, and the CHAARTED and LATITUDE criteria were associated with PFS (Table 3). Age, nadir PSA, hemoglobin, albumin, lactate dehydrogenase, alkaline phosphatase, EOD, and the CHAARTED and LATITUDE criteria were associated with OS (Table 4). Multivariate analysis using Cox’s proportional hazards analysis revealed that the nadir PSA value was an independent predictor of PFS and OS. Hemoglobin, albumin, alkaline phosphatase, EOD, liver metastasis, and the LATITUDE criteria were also independent predictors of PFS while hemoglobin, lactate dehydrogenase, alkaline phosphatase, and the LATITUDE criteria were associated with OS.

**Table 3:** Univariate and multivariate analysis of progression-free survival P< 0.05 was considered statistically significant. PSA, prostate-specific antigen; EOD, extent of disease; CHAARTED, chemohormonal therapy versus androgen ablation randomized trial for extensive disease in prostate cancer

Parameters	Cut-off	Univariate		Multivariate	
		HR (95% CI)	p-value	HR (95% CI)	p-value
Age	<65 vs ≥65 yrs	0.090 (-0.19 – 0.16)	0.92	0.014 (-0.18 – 0.19)	0.88
Initial PSA	<300 vs ≥300 ng/ml	-0.22 (-0.34 – -0.11)	<0.0001	0.063 (-0.060 – 0.19)	0.32
Nadir PSA	<0.036 vs <0.21	1.09 (0.49 – 1.64)		1.28 (0.74 – 1.80)	
	<0.10 vs <0.21	0.40 (-0.20 – 1.02)		0.30 (-0.27 – 0.87)	
	<0.21 vs ≥0.21	0.98 (0.60 – 1.41)	<0.0001	0.89 (0.49 – 1.33)	<0.0001
Hemoglobin	>12 vs ≤12 g/dl	0.40 (0.29 – 0.51)	<0.0001	0.34 (0.21 – 0.47)	<0.0001
Albumin	>3.9 vs ≤3.9 g/dl	0.20 (0.089 – 0.31)	0.0004	-0.20 (-0.33 – -0.066)	0.0034
Lactate dehydrogenase	≤250 vs >250 U/l	-0.31 (-0.43 – -0.18)	<0.0001	-0.14 (-0.28 – 0.0022)	0.054
Alkaline phosphatase	≤500 vs >500 U/l	-0.44 (-0.55 – -0.32)	<0.0001	-0.20 (-0.35 – -0.044)	0.011
Gleason score	<9 vs ≥9	0.029 (-0.081 – 0.14)	0.6	-0.00076 (-0.12 – 0.12)	0.99
EOD	<2 vs ≥2	-0.47 (-0.59 – -0.36)	<0.0001	-0.13 (-0.33 – -0.058)	0.18
Lung metastasis	no vs yes	0.0077 (-0.17 – 0.20)	0.93	-0.018 (-0.21 – 0.19)	0.85
Liver metastasis	no vs yes	0.41 (-0.013 – 1.00)	0.059	0.58 (0.092 –1.21)	0.018
CHAARTED		-0.51 (-0.63 – -0.39)	<0.0001	0.052 (-0.19 – 0.30)	0.68
LATITUDE		-0.49 (-0.61 – -0.37)	<0.0001	-0.26 (-0.45 – -0.076)	0.0054

**Table 4:** Univariate and multivariate analysis of overall survival P< 0.05 was considered statistically significant. PSA, prostate-specific antigen; EOD, extent of disease; CHAARTED, chemohormonal therapy versus androgen ablation randomized trial for extensive disease in prostate cancer

Parameters	Cut-off	Univariate		Multivariate	
		HR (95% CI)	p-value	HR (95% CI)	p-value
Age	<65 vs ≥65 yrs	-0.21 (-0.41 – 0.018)	0.031	-0.010 (-0.22 – 0.19)	0.93
Initial PSA	<300 vs ≥300 ng/ml	-0.15 (-0.27 – -0.032)	0.013	0.062 (-0.076 – 0.20)	0.38
Nadir PSA	<0.036 vs <0.21	1.33 (0.67 – 2.0)		1.09 (0.51 – 1.67)	
	<0.10 vs <0.21	0.59 (-1.3 – 0.13)		0.047 (-0.76 – 0.63)	
	<0.21 vs ≥0.21	1.43 (0.94 – 2.0)	<0.0001	1.25 (0.73 – 1.86)	<0.0001
Hemoglobin	>12 vs ≤12 g/dl	0.45 (0.33 – 0.58)	<0.0001	0.36 (0.22 – 0.49)	<0.0001
Albumin	>3.9 vs ≤3.9 g/dl	0.37 (0.25 – 0.49)	<0.0001	0.069(-0.071 – 0.21)	0.34
Lactate dehydrogenase	≤250 vs >250 U/l	-0.32 (-0.46 – -0.18)	<0.0001	-0.18 (-0.234 – 0.020)	0.028
Alkaline phosphatase	≤500 vs >500 U/l	-0.38 (-0.50 – -0.25)	<0.0001	-0.24 (-0.42 – -0.071)	0.0058
Gleason score	<9 vs ≥9	-0.064 (-0.18 – 0.058)	0.3	-0.036 (-0.17 – 0.097)	0.59
EOD	<2 vs ≥2	-0.30 (-0.42 – -0.18)	<0.0001	0.11(-0.10 – 0.32)	0.29
Lung metastasis	no vs yes	-0.017 (-0.21 – 0.19)	0.87	–0.018 (-0.21 – 0.24)	0.95
Liver metastasis	no vs yes	0.18 (-0.25 – 0.77)	0.45	0.35 (-0.14 – 0.98)	0.17
CHAARTED		-0.36 (-0.49 – -0.23)	<0.0001	-0.15 (-0.42 – 0.12)	0.27
LATITUDE		-0.36 (-0.49 – -0.24)	<0.0001	0.0049(-0.22 – 0.21)	0.27
		0.15 (-0.090 – 0.41)		0.71 (0.44 – 1.0)	
		0.044 (-0.24 – 0.34)		0.11 (-0.19 – 0.42)	
		-0.028 (-0.47 – 0.38)		0.23 (-0.22 – 0.65)	
		0.0083 (-0.49 – 0.45)	0.82	-0.056 (-0.57 – 0.40)	<0.0001

## Discussion

The present report is the first to predict the prognosis of individual patients with metastatic HSPC by remission depth. The nadir PSA cut-off value for each TTCR (three, five, seven, and nine years) was determined using ROC curves. The TTCR was able to be predicted using the serum PSA nadir value, i.e., remission depth; as the nadir PSA level decreased, the TTCR tended to increase, with the target nadir PSA value for the seven- and nine-year TTCR being the same, indicating that most patients without progression to CRPC for seven years would be CRPC-free after nine years.

Several, previous reports used nadir PSA 0.2 ng/ml as a cut-off value, but this comes with some limitations in the clinical setting. First, the cut-off value (0.2 ng/ml) merely predicts a good or bad prognosis but cannot predict the specific prognosis. Second, this cut-off value has already been used as one of several prognostic factors in a multivariate model to predict prognosis. In our study, the target value for each TTCR was determined using ROC curves. Our results suggested that the nadir PSA value alone can predict prognosis without using a model with multiple factors because of its high sensitivity, high specificity, and high AUC on ROC analysis.

Some newly developed drugs have been approved for front-line therapy against metastatic HSPC. However, there is as of yet no evidence-based recommendation for choosing among these options [23]. The nadir PSA level thus remains an accurate and reliable prognostic marker and can facilitate choosing an appropriate drug depending on the life expectancy of each patient. It has the additional benefit of being useful in the clinical setting because of its simplicity.

Of the 170 patients with a PSA value < 0.21 ng/ml, 35 (20.6%) failed to achieve 0.1 ng/ml or lower. Similarly, of the 135 patients with PSA < 0.1 ng/ml, 37 (27.4%) failed to achieve PSA < 0.036 ng/ml. These findings indicate the difficulty of lowering the nadir PSA value to extend the TTCR by two years. The newly developed drugs might be able to lower the nadir PSA value and extend the TTCR but may come at a higher cost; androgen receptor-axis-targeted (ARAT) therapies, for example, are ten times as expensive as the vintage hormonal therapies [1,4,5]. Our cohort comprised 413 patients (97.0%) receiving vintage hormonal therapies as their first-line therapy. Of these patients, 39.8, 31.6, and 23.0% were able to achieve a nadir PSA value <0.21, 0.1, and 0.036 ng/ml, respectively, suggesting that vintage hormonal therapy is adequate for some patients with metastatic HSPC. However, the clinical use of the nadir PSA value for predicting the effect of vintage hormonal therapies early in their administration is limited because the median time to nadir PSA was 9.9 months (range: 1.0-57.8 months) or later than the start of ARAT therapy. The effect of delaying ARAT therapy on the clinical efficiency of anti-HSPC treatment is unclear.

Interestingly, low nadir PSA improved both PFS and OS, indicating that the response to the initial treatment for metastatic HSPC can predict the response to subsequent therapies. Recently, Miyake et al. reported no difference between the length of OS in patients after CRPC onset with their TTCR [24]. In other words, TTCR has the greatest impact on OS. It is important to achieve as long a remission period as possible with the initial treatment to improve the OS rate.

Our results are meaningful in two respects. First, the nadir PSA value can serve as a reliable prognostic marker of OS. Second, the choice of an appropriate drug can be made based on OS.

The present study analyzed the prognosis of a very large cohort of patients with advanced cancer. The median OS was 58.8 months and worse than in several, previous studies [25]. The latter, however, had small sample sizes and included patients with a low serum initial PSA level.

The prognosis of metastatic prostate cancer is classified according to several factors. Serum iPSA is considered a poor prognostic factor [8,26,27] although this finding was contradicted by a recent study reporting that serum iPSA is not a prognostic factor in patients with PSA>100 ng/ml [28]. The latter finding is controversial because it was based on a small number of patients. In our cohort, the iPSA level proved to be a poor prognostic factor when using the median cut-off value of 300 ng/ml.

The present study has several limitations. First, it was retrospective and monocentric. Second, most of the patients had received androgen-deprivation therapy as the primary treatment while only a small percentage received ARAT therapy. This is not in line with current treatment trends. To address this issue, the cut-off value should be determined using a cohort receiving ARAT therapy.

## Conclusions

Our study identified the nadir PSA cut-off value for several TTCRs in patients with metastatic HSPC. Low nadir PSA improved both PFS and OS, indicating the response to the initial treatment for metastatic HSPC. Initial treatment for HSPC should be selected to achieve a low nadir PSA as much as possible.

The nadir PSA value alone can predict prognosis and is useful in routine clinical practice because of its simplicity and accuracy. Further research is necessary to determine whether choosing a drug on the basis of the predicted life expectancy can improve treatments.
